# The route of administration influences the therapeutic index of an anti-proNGF neutralizing mAb for experimental treatment of Diabetic Retinopathy

**DOI:** 10.1371/journal.pone.0199079

**Published:** 2018-06-21

**Authors:** Pablo F. Barcelona, Alba Galan, Hinyu Nedev, Yifan Jian, Marinko V. Sarunic, H. Uri Saragovi

**Affiliations:** 1 Lady Davis Institute-Jewish General Hospital; Center for Translational Research, McGill University, Montreal, Quebec, Canada; 2 Department of Pharmacology and Therapeutics, McGill University, Montreal, Quebec, Canada; 3 School of Engineering Science, Simon Fraser University, BC, Canada; 4 Department of Ophthalmology and Integrated Program for Neuroscience, McGill University, Montreal, Quebec, Canada; University of Tasmania, AUSTRALIA

## Abstract

Many neurodegenerative retinal diseases are treated with monoclonal antibodies (mAb) delivered by invasive intravitreal injection (IVT). In Diabetic Retinopathy there is a scarcity of effective agents that can be delivered using non-invasive methods, and there are significant challenges in the validation of novel therapeutic targets. ProNGF represents a potential novel target, and IVT administration of a function-blocking anti-proNGF mAb is therapeutic in a mouse model of DR. We therefore compared invasive IVT to less invasive systemic intravenous (IV) and local subconjunctival (SCJ) administration, for therapy of Diabetic Retinopathy. The IV and SCJ routes are safe, afford sustained pharmacokinetics and tissue penetration of anti-proNGF mAb, and result in long–term therapeutic efficacy that blocks retinal inflammation, edema, and neuronal death. SCJ may be a more convenient and less-invasive approach for ophthalmic use and may enable reduced frequency of intervention for the treatment of retinal pathologies.

## Introduction

The delivery of drugs to the posterior segment of the eye to treat retinopathies and inflammation can be achieved by direct intravitreal injection (IVT), and in some cases by subconjunctival injection (SCJ), or via systemic intravenous injection (IV). Each approach has benefits and challenges. This manuscript examines the potential value of inhibiting proNGF, the ligand for the p75^NTR^ receptor, as a therapeutic approach for Diabetic Retinopathy (DR). We compare the therapeutic efficacy of IVT, IV and SCJ administration of a function–blocking monoclonal antibody (mAb) against proNGF.

DR, the leading cause of blindness, is characterized by early retinal neurovascular dysfunction [1–3] followed by hypoxia and VEGF–mediated pathological angiogenesis (proliferative DR) at later stages. Currently, preventing pathological angiogenesis remains the only treatment [4, 5] for proliferative DR.

In DR, many challenges result in the virtual absence of novel and validated targets that are disease modifying, and lack of novel mechanisms of action. Indeed, for DR, laser photocoagulation, vitreoretinal surgery [6–11] are used, and few drugs are therapeutic such as intravitreal injection of anti-VEGF antibodies, and anti-inflammatories [12]. Although efficient, these routes are invasive, costly and often carry serious complications. Additional targets are still sought for retinopathies [13], especially targets that are different from VEGF, or that act upstream of VEGF [14].

The activation of p75^NTR^ by proNGF is etiological to early disease pathology in DR and vascular pathology [15] and also to glaucoma [16], and other forms of optic nerve damage [17–19]. Inhibition of either or both the receptor p75^NTR^ or the ligand proNGF could yield a novel therapeutic mechanism of action.

Intravitreal (IVT) delivery of anti-proNGF antibody reduced pro-inflammatory agents promoted by p75^NTR^ (TNFα and α_2_M), reduced blood-retina barrier (BRB) breakdown, reduced retinal edema, preserved retinal structure, and prevented ganglion cell neuronal death [15].

For translational medicine it would be valuable to use a less invasive method than IVT to deliver repeated treatments in chronic diseases such as DR or glaucoma. However, very few studies exist comparing IVT, SCJ and IV delivery of mAbs; and none exist for drugs targeting neurotrophic pathways. The route of delivery must account for pharmacokinetic and pharmacodynamics issues, drug stability and biodistribution, and minimal toxicity, while achieving pharmacologically relevant drug concentrations in the retinal tissue. Moreover, a better understanding of retinal delivery methods for mAbs would have wide applications for many mAbs and for many retinal indications. These are the goals of the work reported here.

Comparative studies evaluating three delivery routes of anti-proNGF mAb show that both IV and SCJ methods are less invasive and attain retinal exposure at therapeutic levels in the mouse model of DR, achieving the same efficacy as IVT injections, for all experimental endpoints. IV delivery requires a relatively high dose of mAb given the volume of distribution but affords sustained retinal exposure. SCJ delivery, as a local non-invasive route, requires a relatively low dose which still affords long–lasting therapeutic benefits, without detectable systemic exposure. SCJ delivery represents a promising novel approach for the treatment of DR, and perhaps other chronic retinal pathologies where the proNGF/ p75^NTR^ axis is implicated including glaucoma and age-related macular degeneration.

## Methods

### Animals

All studies adhered to the Association for Research in Vision and Ophthalmology (ARVO) Statement for the Use of Animals in Ophthalmic and Vision Research, and were approved by the McGill University Animal Care Committee (Protocol #2017–7381). C57Bl/6 mice (male, age 10 weeks) were purchased from Charles River Laboratory (St. Zotique, Quebec, Canada). Animals were housed 12 hours dark/light cycle with food and water *ad libitum*. Diabetes was induced by intraperitoneal injection (IP) of streptozotocin (STZ) (60 mg/kg) (Sigma-Aldrich, St. Louis, MO) dissolved in sodium citrate buffer (0.01 M, pH 4.5) on five consecutive days [15]. Age-matched, C57BL/6 mice injected with sodium citrate buffer were used as controls. Blood glucose was measured using a glucometer (Abbott Lab.), and fasting blood glucose levels routinely higher than 17 mmol/L (300 mg/dl) were considered to be diabetic [15]. Mice were weighted on weekly basis.

### Anti-proNGF antibody labeling with biotin

The anti-proNGF mAb was manufactured in house and purified using Protein-G columns [17] and was labeled using the General Sulfo-NHS-LC-Biotin Protein Biotinylation Protocol, from ProteoChem.

### Drug delivery

For pharmacokinetic studies of anti-proNGF mAb in normal animals, we performed IVT injections in the left eye, and SCJ injections in the right eye. Samples were collected at 30 and 60 minutes after IVT or SCJ delivery. In therapeutic studies of diabetic animals, IVT and SCJ anti-proNGF (right eye) or PBS vehicle (left eye) injections were done at 2.5 weeks of disease.

### Intravitreal injections (IVT)

A volume of 2.5 μl containing a total of 2 μg anti-proNGF was slowly delivered into the mouse’s vitreous chamber using a Hamilton syringe, and confirmed microscopically. After the injection, the syringe was left in place for 30 seconds and slowly withdrawn to prevent efflux [15].

### Subconjuctival injections (SCJ)

A volume of 20 μl containing a total of 20 μg anti-proNGF, or corresponding volumes of vehicle (PBS) were injected as a depot. The conjunctiva was gently pulled from the sclera using tweezers. Half of the total volume of anti-proNGF was delivered into the superior subconjunctival space and the other half into the nasal subconjunctival region, using a microsyringe with a 33G needle.

### Intravenous tail vein injection (IV)

A volume of 100 μl containing a total of 100 μg anti proNGF antibody, or corresponding volumes of vehicle (PBS) were injected in the peripheral tail vein with a 33G needle, using a restraining device.

### Western blot

Eyes were enucleated and retinas immediately dissected and placed into protein lysis buffer (20 mM Tris, pH 7.5; 137 mM NaCl; 2 mM EDTA pH 8; 1% Nonidet P-40; phosphatase inhibitor cocktail, Roche #04 906 837 001; and protease inhibitor cocktail, Roche # 04 693 159 001) and homogenized. Samples were centrifuged and 30 μg/lane was separated on an SDS-PAGE gel (8–12%) and electro-blotted onto PVDF membranes (BioRad). After blocking steps with 2% bovine serum albumin (BSA) in T-TBS (2% Tween-20; 20 mM Tris and 150 mM NaCl), membranes were incubated overnight at 4°C with mouse antibody to β-actin (sc-47778, Santa Cruz Biotechnology; 1:1000), rabbit antibody to Tumor Necrosis Factor-α (AB2148P, Millipore; 1:2,000), rabbit antibody to α_2_M (sc-8517, Santa Cruz Biotechnology; 1:2,000) and biotinylated anti-proNGF (1:1,000). After washing, membranes were incubated with secondary for 2 hr at room temperature (1:10,000 HRP conjugated anti-mouse (A9044, Sigma) or anti-rabbit antibodies (A0545, Sigma) or 1:10,000 horseradish peroxidase-conjugated avidin (A3151, Sigma). Membranes were imaged with LAS-3000 imager (FujiFilm) and bands were assessed using densitometry plugins in ImageJ 1.47 software. Normalization of the data (total n = 3 independent experiments, each in triplicate) was done using the optic density of the band for each target protein divided by the optic density of α-actin. One-way analysis of variance (ANOVA) followed by Bonferroni post-hoc analysis was applied. Control healthy values were always statistically different from diabetic untreated mice. Then, all “raw” values from diabetic animals were normalized to the control healthy group, which was assigned the arbitrary value “1”. The kDa reported are from M_r_ calculated using standard markers, as well as positive control proteins (proNGF, NGF, TNFα and α_2_M) resolved in the same gel in parallel lanes.

### Optic coherence tomography (OCT) imaging

A spectrometer-based FD-OCT system was used to acquire the retinal images. FD-OCT is a noninvasive method that allows time-kinetic studies in the same animal, with axial resolution in tissue nominally <3 μm and repeatability of the measurements from B-scans <1 μm. Data acquisition was performed using custom software written in C++ for rapid frame grabbing, processing, and display of two-dimensional images [20, 21]. Manual segmentations were used to measure the thicknesses of the mice retinas as described [22]. During scanning, three volumes were acquired in different sectors of the retina, using the ON head as landmark. After processing, three B-scans were randomly selected from each volume and retinal thickness analyses were performed with Image J software. In each B-scan, the thickness of the nerve fiber layer–ganglion cell layer (GCL)–inner plexiform layer (IPL), hereafter referred to as NGI [22]. Data are shown as average each thickness in μm ± SEM (absolute values) in control *versus* diabetic injected with either vehicle (PBS) or with anti-proNGF, n = 3 independent experiments, 3–4 mice per group.

### Immunohistochemistry

Whole eyes were enucleated from mice and fixed in 4% PFA at room temperature for 2 h. Eyes were saturated overnight at 4°C in a 30% sucrose solution prior to immersion in optimum cutting temperature compound (cat #4583, Sakura). Retinal cross sections of 10 μm (Leica Cryostat) were subsequently washed with PBS, permeabilized for 60 min at room temperature with 3% BSA, 0.2% Triton X-100 and 0.05% Tween20 in PBS, and stained with primary antibodies mouse anti-CRALBP (15051, Abcam; 1:1000), rabbit anti-Tumor Necrosis Factor-α (AB2148P, Millipore,1:1000), rabbit anti-α_2_M (sc-8517, Santa Cruz; 1:1000). Labelled secondary antibodies goat anti-mouse IgG Alexa Fluor 594 (A11020, Life Technologies) and goat anti-rabbit IgG Alexa Fluor 488 (A11034, Life Technologies) were used. All antibodies were prepared with 1% BSA and 0.3% Triton X-100 in PBS. Images were obtained using an IX81 confocal microscope (Olympus) equipped with Fluoview 3.1 software (Olympus). As immunostaining controls, adjacent tissue sections were processed equally but without primary (without rabbit anti-TNFα or rabbit anti-α_2_M or mouse anti-CRALBP), followed by the proper secondary (goat anti-mouse IgG Alexa Fluor 594 or goat anti-rabbit IgG Alexa Fluor 488, both 1:800). In all cases background levels were undetectable. For each experimental condition, a minimum of 6 images were acquired from 3 sections cut from different areas of the retina (n = 3 retinas per group). Data are shown as α_2_M or TNFα area values (± SEM) in diabetic animals treated with vehicle or anti-proNGF normalized to healthy control (arbitrary value “1”).

### Fluorescence “In situ” hybridization (FISH)

The Digoxigenin RNA labeling kit (#11175025910, Roche) was used to generate the DIG-labeled probes. Efficient labeling was verified in agarose gels (1%). After enucleation, the eyes were immersed overnight in fixative at 4°C (4% paraformaldehyde (PFA) in PBS pH 7.4), followed by cryoprotection in 30% sucrose overnight at 4°C. Eyes were embedded in optimum cutting temperature compound (#4583, Sakura) and frozen with dry ice. Cryostat-cut sections (20 μm thick) were mounted onto gelatin-coated glass slides, re-fixed in 4% PFA, permeabilized with proteinase K and acetylated. Hybridization was carried by incubating the slides with either 200 ng/ml of DIG-labeled TNFα or α_2_M antisense RNAs probes overnight at 72 ^0^C in a hybridization oven (Robbins Scientific). As controls, hybridization with either 200ng/ml of DIG-labeled TNFα or α_2_M sense RNAs probes was performed on parallel slides using the same experimental conditions. Hybridization was followed by non-stringent and stringent washes, and incubation with anti-DIG-HRP (#11 207 733 910, Roche, 1:1000) overnight at 4 ^0^C. The amplification reaction was performed using the TMR-conjugated TSA kit (NEL744001KT, Perkin Elmer) following the manufacturer’s protocol. Finally, sections were washed and cover-slipped using Vectashield mounting media with DAPI (H-1500, Vector Laboratories). Images were obtained using an IX81 confocal microscope (Olympus) equipped with Fluoview 281 3.1 software (Olympus). For each experimental condition, a minimum of 8 images were acquired from 3 sections cut from different areas of the retina (n = 3 retinas per group). Data are shown as α_2_M or TNFα area values (± SEM) in diabetic animals treated with vehicle or anti-proNGF normalized to healthy control (arbitrary value “1”).

### Vascular permeability assay

Evans blue permeation was analyzed by measuring albumin-Evans blue complex leakage from retinal vessels as previously described (Ma et al., 1996). Briefly, animals were injected intravenously with a solution of Evans blue (2% wt/vol dissolved in PBS). After 48h, animals were sacrificed, the whole eyes enucleated, immediately embedded in OCT compound and cross sections were prepared. Images were obtained using an IX81 confocal microscope (Olympus) equipped with Fluoview 281 3.1 software (Olympus) using identical exposure time, brightness, and contrast settings. For each experimental condition, at least 6 images were acquired from 3 sections cut from different areas of the retina (n = 3 retinas per group). The area of the Evans blue permeation was measured using ImageJ software; an arbitrary rectangle that included all layers of the retina (GCL, IPL. INL, ONL, PhR and RPE) was drawn and the area in pixels quantified. For each experimental condition, a minimum of 8 images were acquired from 3 sections cut from different areas of the retina (n = 2 retinas per group). Data are shown as Evans Blue area values (± SEM) in diabetic animals treated with vehicle or anti-proNGF normalized to healthy control (arbitrary value “1”).

### Statistical analysis

Results are presented as mean ± SEM for all studies. One-way ANOVA with significance α = 0.05 or higher were used for processing data. Bonferroni post-hoc analysis was used for calculating significance between groups. Two-tailed student t-tests were used to test for significance between two means.

## Results

### Intravenous administration of anti-proNGF antibody does not cause systemic toxicity

An anti-proNGF mAb binds to and neutralizes proNGF [23]. To evaluate intravenous (IV) dosing, the mAb or control vehicle was administered in normal or diabetic mice at 2.5 weeks after the induction of diabetes (S1A Fig). Blood samples were collected at week 6 after the onset of diabetes. Anti-proNGF treatment did not show any effect in the levels of blood glucose in diabetic mice compared to control diabetic littermates. There were no significant changes in the levels of urea nitrogen or amylase, in any of the groups, at the experimental endpoint (Table 1). Longitudinal analysis of the body weights showed no net weight gain or loss (S1B Fig). The mice exhibited no change on cage activity or the condition of their fur, no pain behavior (vocalization, lack of grooming, limited mobility, loss of muscle tone), or observable negative side effects. These data indicate that IV injections of this anti-proNGF mAb are safe. However, one negative aspect is that this route requires larger quantities of product given the large systemic volume of distribution.

**Table 1:** Toxicity studies in diabetic mice treated with systemic anti-proNGF antibody.

Treatment	Glucose (mmol/L)	BUN urea (mmol/L)	Amylase (U/L)
Normal values	5.0–10.7	6.4–10.4	1691–3615
**STZ**	19.24 ± 2.4	7.25 ± 0.1	2992 ± 11
**STZ+anti-proNGF (IV)**	21 ± 3.9	7.70 ± 0.6	2754 ± 12

Toxicity studies in mice 6 weeks after the onset of diabetes comparing diabetic untreated or diabetic treated with anti-proNGF mAb, versus control naïve normal mice. No difference was observed in blood glucose levels between the two diabetic groups. No alterations were detected in blood urea nitrogen (BUN) or amylase levels at the experimental end point.

### Detection of anti-proNGF mAb in the eye after intravenous (IV), intravitreal (IVT) or subconjunctival (SCJ) administration

The mAb was tagged with biotin to assess its biodistribution. Quality control assessment of anti-proNGF•biotin by western blots using Avidin-HRP as secondary detected a single 150 kDa band under non-reducing conditions (intact biotinylated antibody) and two bands of 50 and 25 kDa under reducing conditions (heavy and light chains) (S2 Fig).

**Fig 1:**
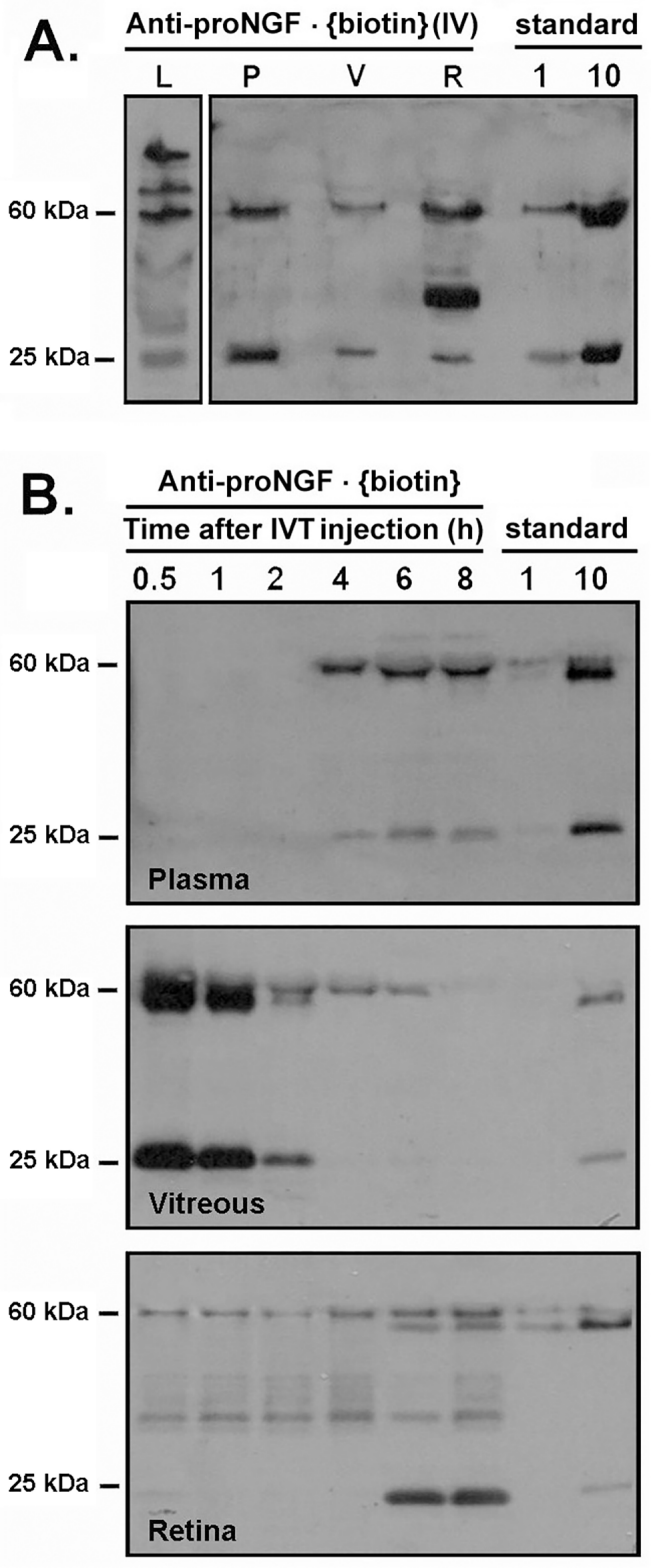
Detection and pharmacokinetics of anti-proNGF antibody in vitreous humor, retina and plasma, after systemic administration. **(A)** Western blot of samples of liver (L), vitreous (V), plasma (P) and retina (R) two days after IV injections of 100 μg anti-proNGF•biotin mAb. **(B)** Pharmacokinetics of anti-proNGF•biotin mAb by Western blot of samples collected at the indicated time points after IVT injection of 2 μg mAb (n = 3 mice per group). For all Western blots, 10μl plasma, 2 μl vitreous, and 20 and 10 μg total protein from retina and liver, respectively, were loaded. As standard controls, 1 and 10 ng of anti-proNGF•biotin mAb protein are shown.

In healthy adult mice, the biodistribution of the anti-proNGF•biotin mAb (100 μg IV dose) was analyzed by collecting tissues 2 days after administration. The anti-proNGF•biotin mAb was detected in liver, plasma, vitreous and retina (Fig 1A). In addition to the expected bands, in liver there are larger size products that suggest mAb modifications such as glucuronidation, and in retina there is an intermediate size product at ~30 kDa that suggests degradation possibly after internalization of proNGF/anti-proNGF mAb complexes (which have been shown previously [23]. The data shows that the anti-proNGF antibody can reach the vitreous and the retina after systemic injection in healthy adult mice and is likely able to pass through the blood-retina-barrier (BRB).

**Fig 2:**
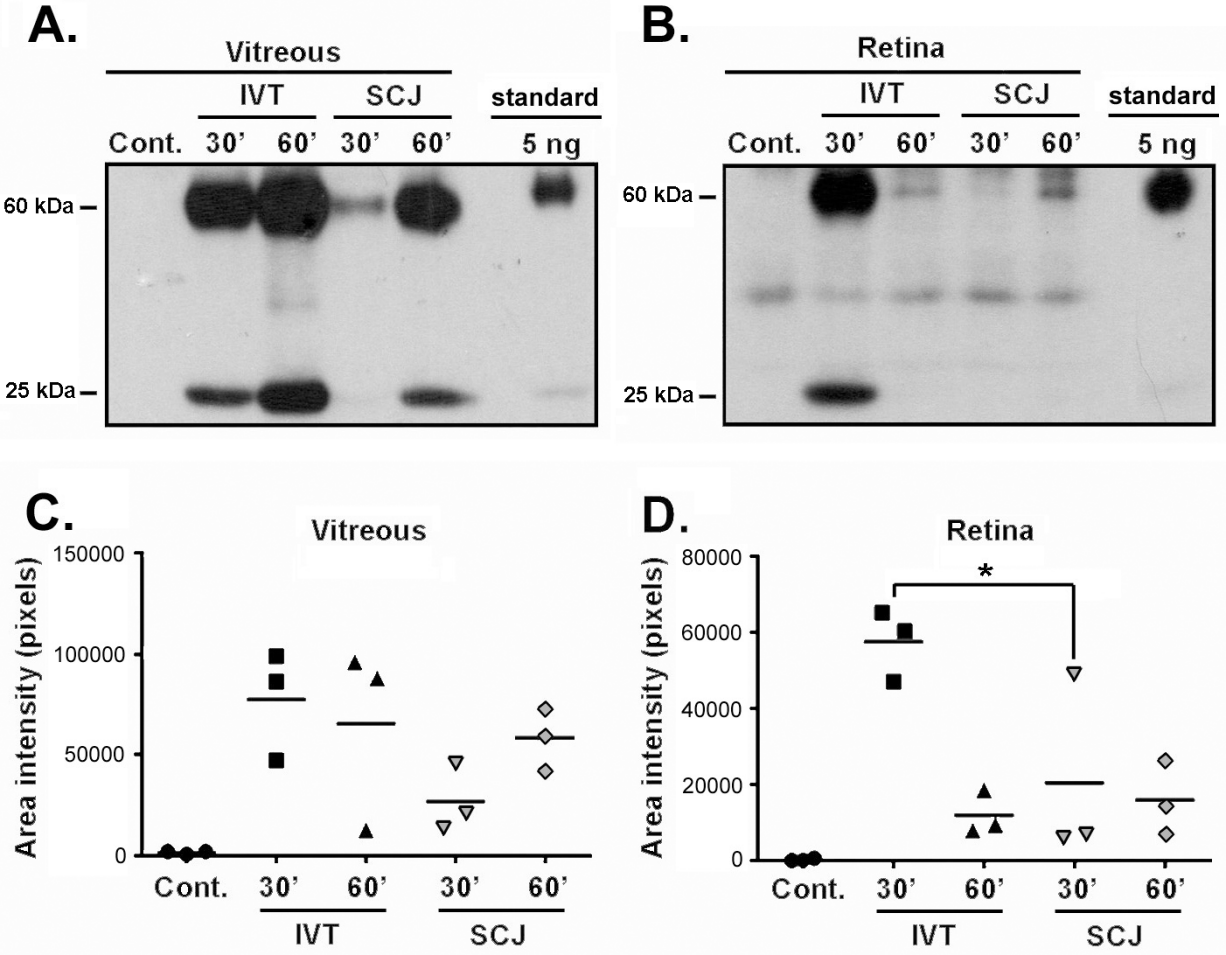
Detection and pharmacokinetics of anti-proNGF antibody in vitreous humor and retina after local administration. Analysis of the pharmacokinetics of biotinylated anti-proNGF mAb by Western blot in **(A)** vitreous and **(B)** retina, at the time points indicated after IVT or SCJ injection of 2 μg or 20 μg of anti-proNGF•biotin mAb. As standard controls, 5 ng of anti-proNGF•biotin mAb are shown. For retina 1/15 of the whole retina sample was loaded per lane; and for vitreous 1/3 of the total sample was loaded per lane. **(C-D)** Quantification of the anti-proNGF mAb in vitreous and retina at 30 and 60 min after IVT and SCJ injections (one-way ANOVA, followed by Bonferroni post-hoc analysis, * p< 0.05, in IVT *versus* SCJ retina samples at 30 min post-injection; n = 3 mice per group).

Analysis of the biodistribution after IVT injection showed a time-dependent decrease of anti-proNGF•biotin mAb in the vitreous (the site of administration), and a corresponding time-dependent increase in the retina (between 1–10 ng, 30 min after injection). There was detectable anti-proNGF•biotin mAb in plasma 4 hour after injection, indicating diffusion from the vitreous into systemic circulation (Fig 1B). A comparative pharmacokinetic study of the anti-proNGF•biotin mAb in the retina was done after IVT (2 μg) or SCJ (20 μg) injections (Fig 2). The concentration was significantly more elevated in the retina after IVT compared to SCJ administration at the 30 min time point (p<0.05, n = 3) (Fig 2D). This is due most likely to the slower release from the subconjunctival depot. However, both the IVT and the SCJ routes afford similar concentrations of anti-proNGF (>5 ng) in the vitreous humor (Fig 2A and 2C**)**, and in the retina (Fig 2B and 2D) at the 60 min time point.

Together, these data indicate that IV, IVT and SCJ routes can be used, and that each route requires a different dose of anti-proNGF mAb to achieve a desired ~5 ng concentration in retina; with IVT and SCJ routes requiring the least amounts. In addition, IVT (and of course IV) routes result in detectable systemic exposure to mAb which may not be desirable from a regulatory perspective. This is likely due to the bolus IVT mAb entering systemic circulation either by diffusion into the aqueous humor (anterior route); or thorough permeation through the blood-retinal barrier (posterior route). In contrast, the SCJ route delivers the mAb locally with minimal systemic exposure. This is likely due to the slower release from the local SCJ depot resulting in slower diffusion and higher dilution rates.

### Intravenous (IV) and subconjunctival (SCJ) injection of anti-proNGF mAb in diabetes preserves retinal structure

Previously, we demonstrated proNGF upregulation in the retina, ~3 weeks after the onset of diabetes [15]. To test whether systemic administration of anti-proNGF antibody had a protective effect against retinal neurodegeneration, mice were injected with 100 μg of anti-proNGF into the tail vein (IV) at 2.5 weeks after the induction of diabetes. Retinal structures were quantified in longitudinal studies by optic coherence tomography (OCT), a non-invasive technique. OCT was performed from week 3 to week 6 of diabetes, measuring the Nerve fiber layer (NFL), the Ganglion cell layer (GCL) and the Inner plexiform layer (IPL), herein termed NGI, allowing us to estimate the density of the RGC cell bodies and fibers [17].

**Fig 3:**
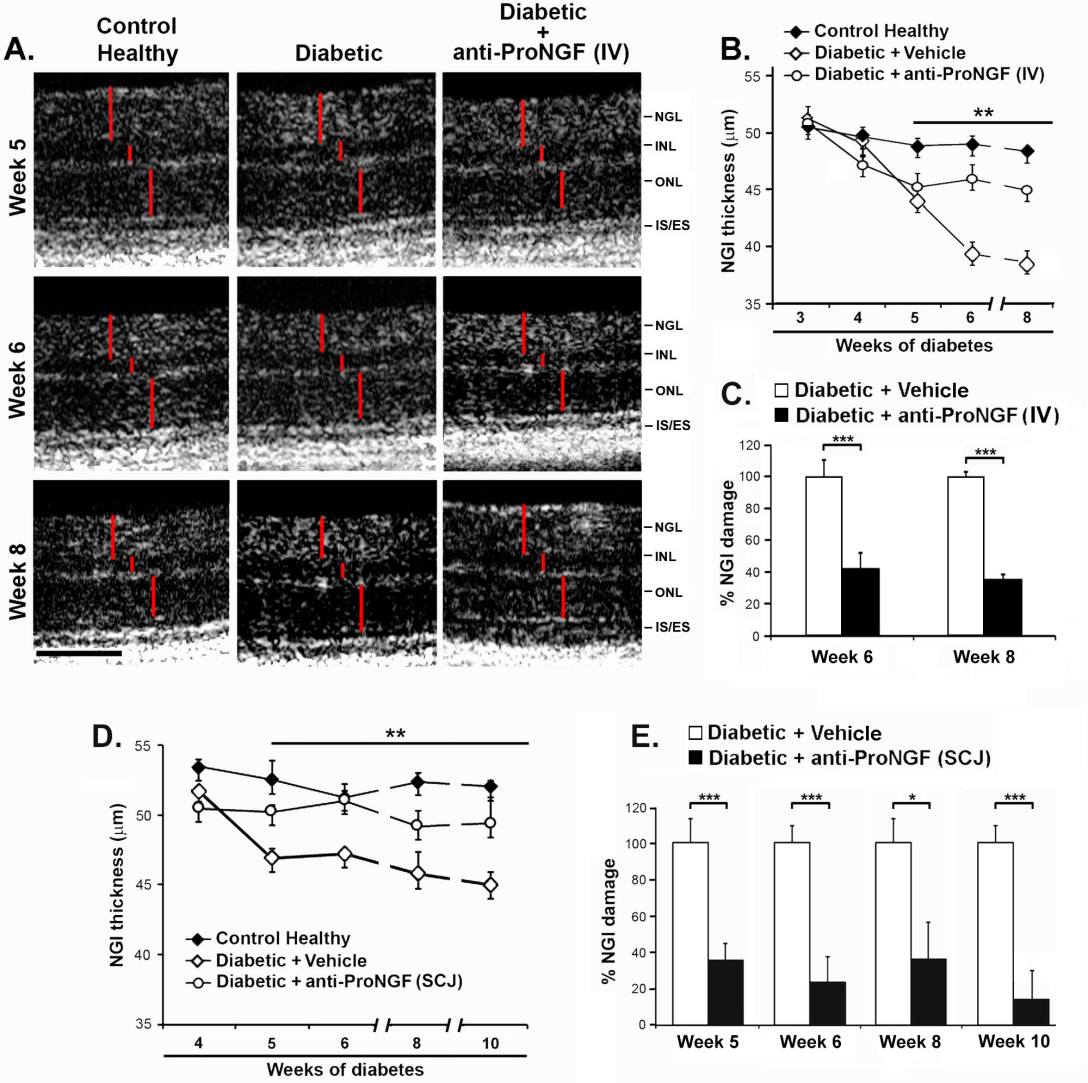
Systemic and subconjunctival delivery of anti-proNGF mAb protects retinal structure. **(A)** Representative sections of B-scans OCT images from diabetic mice (at 5, 6 and 8 weeks of diabetes) ± treatment, one-time injection of anti-proNGF mAb IV (100 μg bolus dose) at week 2.5 of diabetes; compared to age-matched control healthy animals. Vertical red lines delimit the thickness of the retinal layers. Scale bar 100 μm. NGI: the neuronal structure comprising Nerve fiber layer-Ganglion cell layer-Inner plexiform layer; INL: inner nuclear layer; ONL: outer nuclear layer; IS/ES: internal/external segments. **(B)** Histogram of time-dependent changes in NGI thickness ± SEM (n = 3 independent experiments with total n = 9 mice per group). Anti-proNGF IV significantly protected the NGI structure from week 6 to at least week 8 (p<0.001). **(C)** Percent of NGI damage relative to vehicle-treated diabetic retinas (100% NGI damage) at weeks 6 and 8 after onset of diabetes. Systemic treatment significantly reduced NGI damage compared to diabetic vehicle-treated retinas (*** p< 0.001, n = 3 independent experiments, total of 9 mice per group up to week 6; n = 2 mice per group at week 8). **(D)** Time-dependent changes in NGI thickness ± SEM in mice with 4, 5, 6, 8 and 10 weeks of diabetes ± treatment, one time injection of anti-proNGF mAb SCJ (20 μg) at week 2.5 of diabetes; compared to age-matched control healthy animals (2 independent experiments, n = 8 mice per group). SCJ treatment significantly protected the NGI structure at week 10 (p<0.05). All statistical analysis (panels A-D) were one-way ANOVA with significance established at α<0.05, followed by Bonferroni’s correction for multi-comparisons. **(E)** Percent of NGI damage relative to diabetic vehicle-treated retinas (100% NGI damage) at weeks 5, 6, 8 and 10 after the onset of diabetes. SCJ treatment significantly reduced NGI damage compared to diabetic vehicle-treated retinas at both time points (t-test, *** p< 0.001, * p<0.05, 2 independent experiments, n = 6 mice per group up to week 8; n = 3 mice per group at week 10).

Treatment of diabetic mice with IV anti-proNGF significantly protected against NGI thinning (46.33 ± 1.12 μm compared to 39.93 ± 1.14 μm in age-matched diabetic non-treated mice; p<0.001, n = 9, after 6 weeks of diabetes) (Fig 3A and 3B). The protective effect achieved by IV administration of anti-proNGF mAb corresponds to ~60% at week 6 (43.56 ± 9.90%NGI damage in diabetic-treated mice *versus* age-matched diabetic non-treated mice, p<0.001, n = 9) and ~70% at week 8 (n = 2) (Fig 3C). There were no alterations of the INL or ONL retinal layers of any group, and these layers were quantified as internal controls (data not shown). Note that anti-proNGF mAb was administered IV, once, at week 2.5 after induction of diabetes, and that therapeutic efficacy was maintained up to week 6 of diabetes (e.g. for at least 3.5 weeks).

Similar therapeutic studies were done using the SCJ delivery route. OCT was performed longitudinally from 4 to 10 weeks following the onset of diabetes. Treatment of diabetic mice with a single SCJ injection of 20 μg anti-proNGF mAb at 2.5 weeks after induction of diabetes affords a significant protective effect from week 5 to week 10, compared to each age-matched diabetic non-treated mice; p<0.01, n = 9) (Fig 3D). Setting the NGI damage in vehicle-treated diabetic eyes as maximal (100%), the protection of the NGI layers in diabetic eyes was ∼60% at week 5, ∼70% at week 6, ∼60% at week 8 (p < 0.001, or p< 0.05; n = 9) and ∼80% at week 10 (p<0.001, n = 3) (Fig 3E).

### Reduced neurotoxic factors in diabetic retina after intravenous (IV) and subconjunctival (SCJ) administration of anti-proNGF antibody

We previously demonstrated that increased levels of proNGF protein lead to increased inflammatory cytokines α_2_M and TNFα in diabetes [15]. Thus, we investigated this pathway after administration of anti-proNGF antibody by IV or SCJ routes.

**Fig 4:**
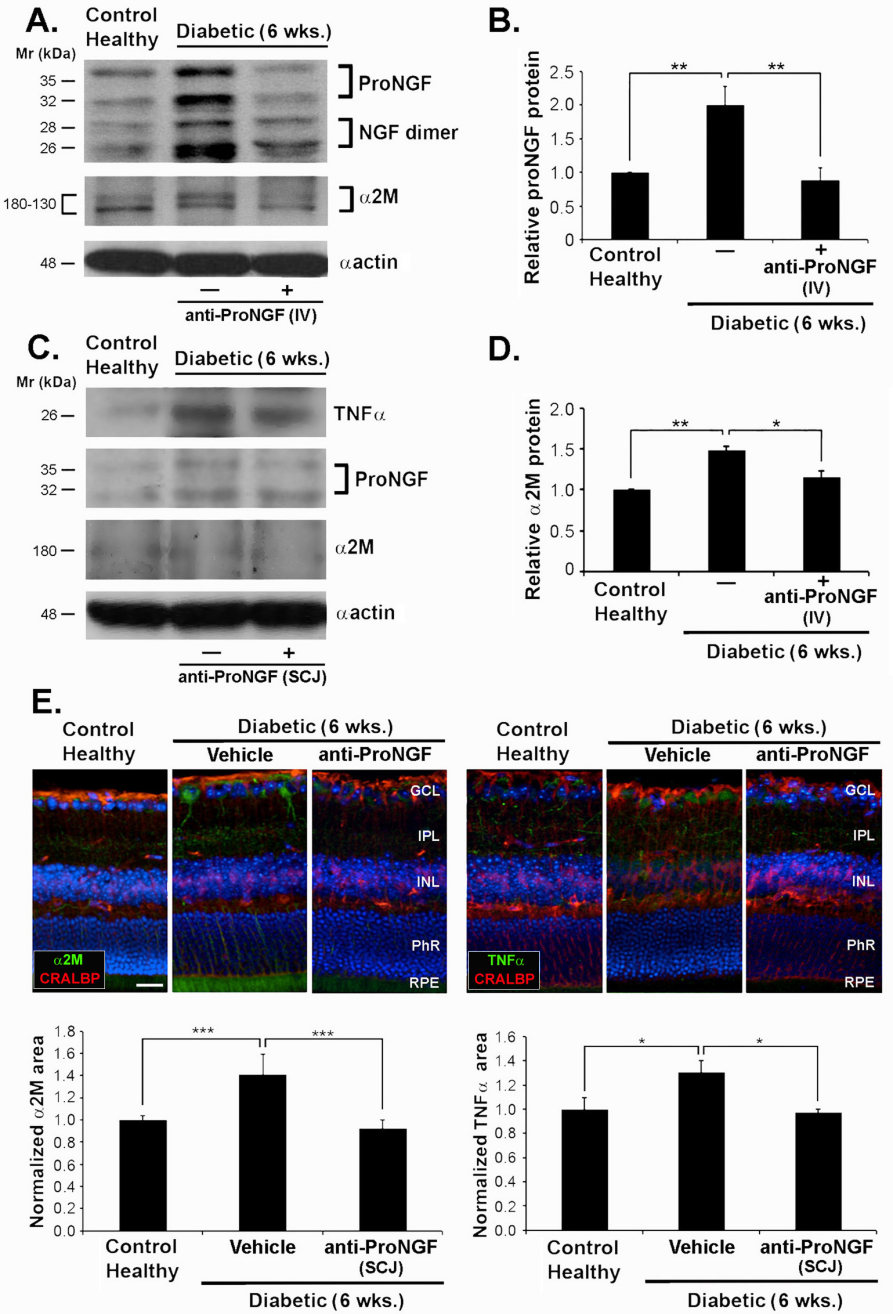
The increase of cytotoxic factors in diabetes is prevented by systemic and subconjunctival administration of anti-proNGF mAb. Protein expression in whole retina analyzed by Western blot of 6-week-diabetic mice ± treatment with injection of anti-proNGF at week 2.5 of diabetes; compared to age-matched control healthy mice. **(A)** α_2_M and proNGF expression after IV injection of anti-proNGF mAb. The IV treatment prevented the increase of α_2_M and inhibited the increase of proNGF. **(C)** α_2_M, TNFα and proNGF expression after SCJ injection of anti-proNGF at week 2.5 of diabetes. The SCJ treatment prevented the increase of TNFα, α_2_M, and proNGF. **(B, D)** Quantification of Western blot data, * p< 0.05, ** p < 0.01, versus healthy animals (n = 6 animals per group). **(E)** Confocal microscopy images of retina sections of 6-week diabetic mice ± treatment with SCJ injection of anti-proNGF or vehicle at week 2.5 of diabetes, compared with age-matched naive control animals. Localization of α_2_M (green) is in the GCL, whereas TNFα (green) is around the end-feet of CRALBP-positive (red) Muller cells on the GCL. Nuclei were counterstained with DAPI. The expression of both TNFα and α_2_M was reduced on diabetic mice treated with anti-proNGF. Data are normalized to TNFα and α_2_M area values (± SEM) relative to healthy control. A total of 10 images at 20X magnification were taken from n = 3 retinas per group, *p < 0.05; ***p<0.001. One-way ANOVA with significance established at α<0.05, followed by Bonferroni’s correction for multi-comparisons was used. Scale bar, 25 μm. NFL, nerve fiber layer; GCL, ganglion cell layer; IPL; inner plexiform layer; INL, inner nuclear layer; PhR, photoreceptor layer; RPE, retinal pigment epithelium.

Treatment with anti-proNGF by IV injection in diabetic mice normalized the increased levels of proNGF protein (0.876 ± 0.19 in treated mice compared to 1.96 ± 0.29 in control diabetic mice, p<0.01, n = 3) (Fig 4B) and the levels of α_2_M protein (1.16 ± 0.07 compared to 1.48 ± 0.05 in control diabetic mice, p<0.05, n = 3) (Fig 4D) in the retina at 6 weeks after the induction of diabetes (Fig 4A**)**. Similar normalization of TNFα and α_2_M proteins was quantified after SCJ injection of anti-proNGF at week 6 after the onset of the disease (Fig 4C).

The normalization of inflammatory cytokines after SCJ antibody administration was confirmed by immunohistochemical analysis of retinal sections. We observed a significant attenuation in α_2_M protein in the RGC layer (Fig 4E), as well as the TNFα protein localized around the end-feet of CRALBP-positive Muller glial cells and in the RGC layer (Fig 4E).

**Fig 5:**
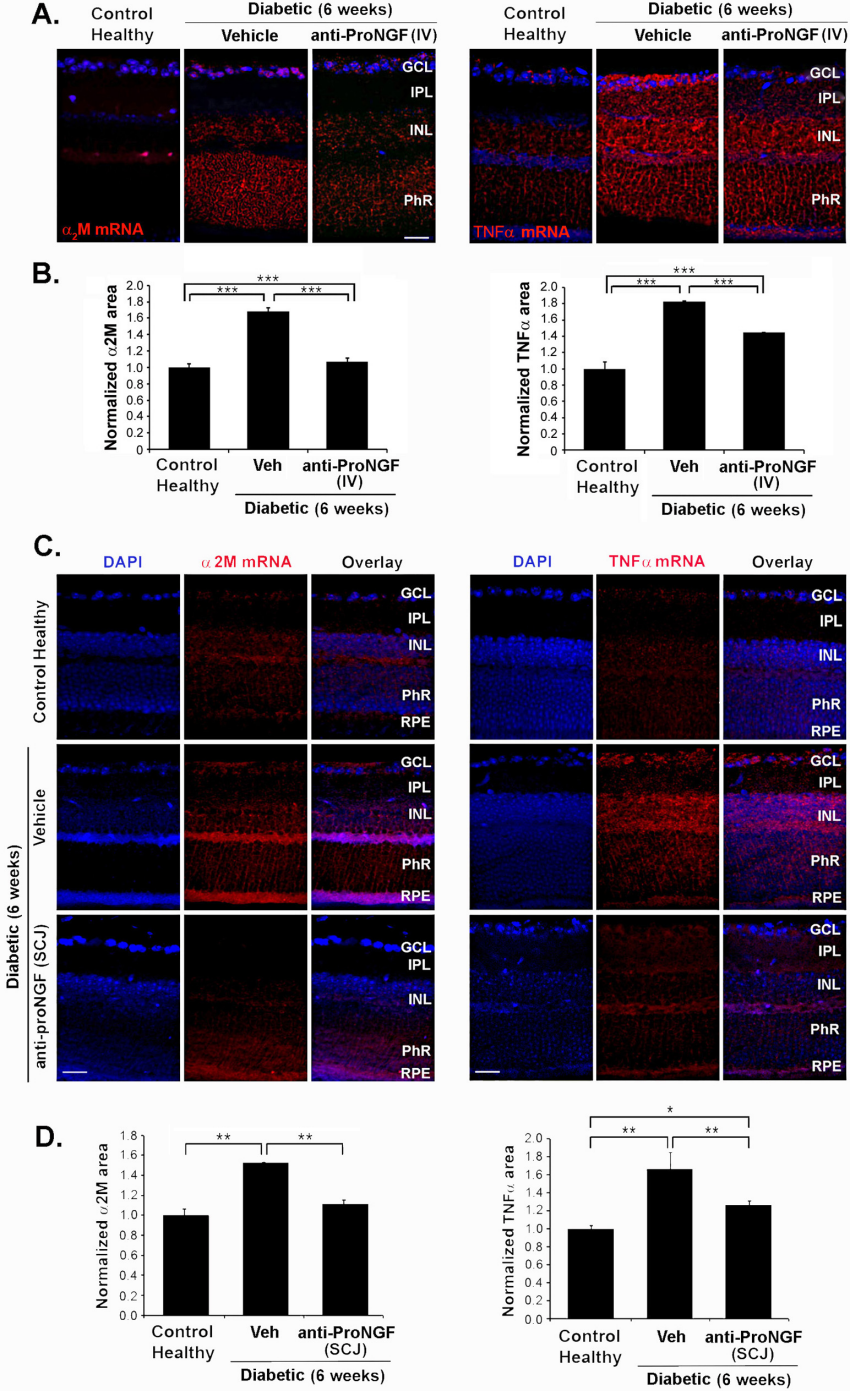
Systemic and subconjunctival administration of anti-proNGF mAb in diabetes reduces the mRNA levels of cytotoxic factors in the retina. Fluorescence *in situ* hybridization (FISH) show the distribution and the induction of α_2_M or TNFα mRNAs, probed with DIG-labeled α_2_M or TNFα antisense RNAs (red) in mice retina after 6 weeks of diabetes ± treatment with IV injection **(A, B)** or with SCJ injection **(C, D)** at week 2.5 of diabetes; compared to age-matched control healthy animals. Nuclei are counterstained with DAPI. Images are representative of 3 independent experiments. Scale bar 25 μm. **B** and **D** are quantification of α_2_M and TNFα mRNAs in diabetic relative to control healthy in all retinal layers. Data are shown as average of normalized area values (±SEM) relative to healthy control. Anti-proNGF significantly decreased α_2_M and TNFα mRNAs compared to diabetic-vehicle untreated retinas. *** p< 0.001 and ** p< 0.01. n = 3 independent experiments each with 2 animals/group. One-way ANOVA with significance established at α<0.05, followed by Bonferroni’s correction for multi-comparisons GCL: ganglion cell layer; INL: inner nuclear layer; OPL: outer nuclear layer; PhR: photoreceptor layer; RPE, retinal pigment epithelium.

Since α_2_M and TNFα are soluble factors that can originate from outside the retina, we examined their mRNA expression by *in situ* mRNA hybridization on retinal sections of diabetic mice at 6-weeks after STZ injection. The results show a significant increase of α_2_M (p< 0.001) and of TNFα (p<0.001) (n = 3) mRNAs (Fig 5B), compared to control healthy retinas. The up-regulated α_2_M and TNFα mRNAs were significantly normalized in diabetic animals treated with anti-proNGF mAb delivered by either IV or SCJ (p<0.001 and p<0.01, n = 2) (Fig 5A–5D).

Together, these results suggest that the anti-proNGF mAb administrated either systemically IV or locally SCJ afford a protective effect comparable to the IVT route [15], protecting the retinal structure by inhibition of inflammatory cytokines produced locally in the diseased retina.

### Vascular permeability is attenuated by systemic or subconjunctival administration of anti-proNGF mAb in diabetes

The pathophysiology of diabetes is characterized by a cascade of events that include loss of brain-retinal barrier function causing retinal edema.

**Fig 6:**
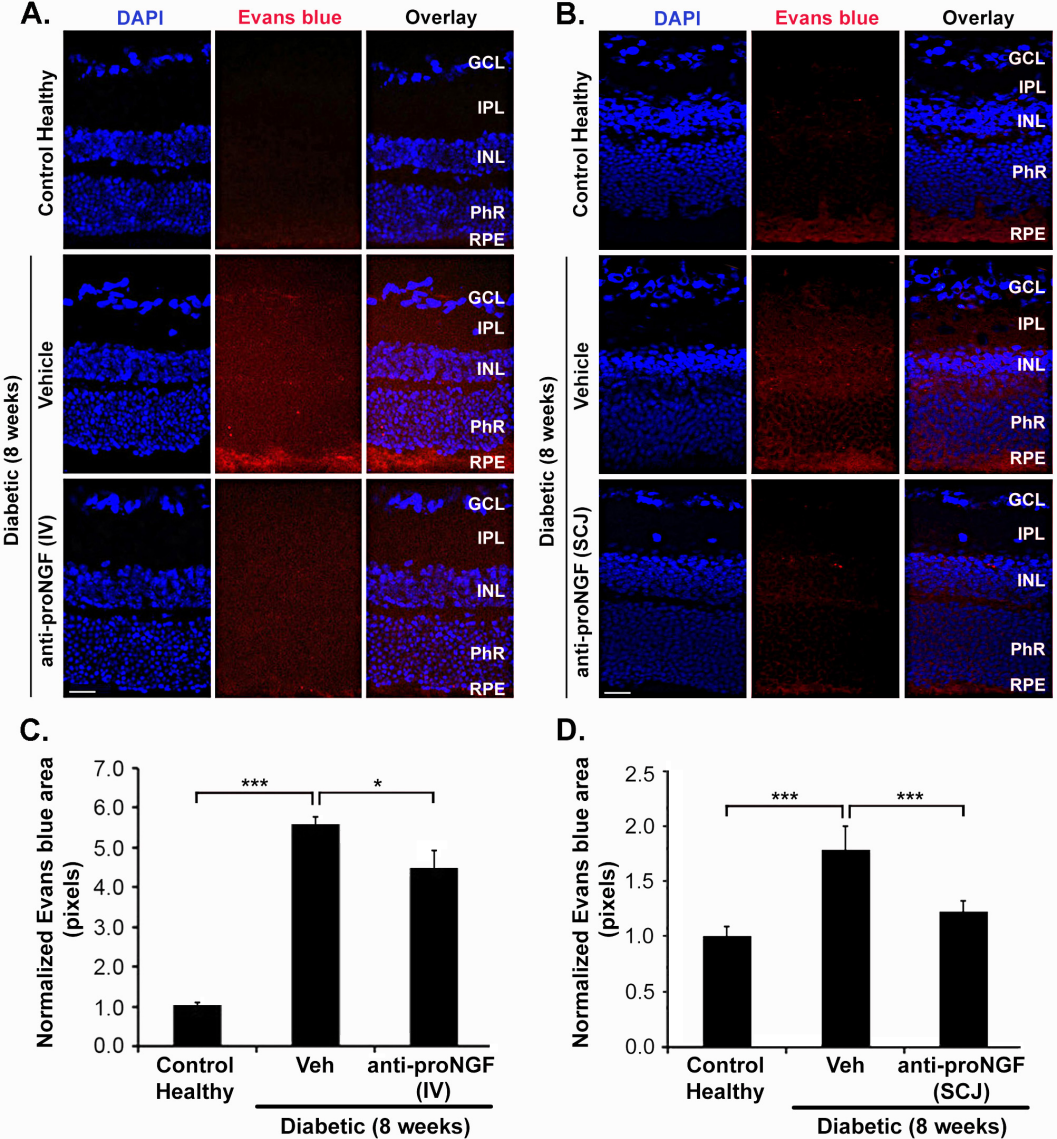
Systemic and subconjunctival administration of anti-proNGF mAb in diabetes reduces pathological vascular permeability in diabetes. Confocal images of retinal sections from 8-week-diabetic mice ± treatment with anti-proNGF mAb at week 2.5 of diabetes. **(A)** IV injection (n = 2 mice) **(B)** SCJ injection (n = 3 mice); compared to age-matched healthy controls or diabetic non-treated mice (n = 3). Red signal depicts leakage of Evans blue into the retina. Nuclei are counterstained with DAPI. Scale bar 25 μm. **(C, D)** Data are showed as normalized Evans Blue area (± SEM) relative to control healthy mice. Treatment by either the IV or the SCJ route reduces plasma extravasation). One-way ANOVA with significance established at α<0.05, followed by Bonferroni’s correction for multi-comparisons, *** p< 0.001, * p< 0.05. A total of 10 images were taken from each retina. Images were taken at 20X.

We analyzed the effect of either IV or SCJ delivery of anti-proNGF mAb, versus vehicle control group, on plasma extravasation at 8 weeks of diabetes. Analyses of retinal sections showed that anti-proNGF treatment by the IV or SCJ routes caused a decrease in vascular leakage (4.5 ± 0.45 or 1.2 ± 0.10) compared to diabetic non-treated mice (5.6 ± 0.20 or 1.8 ± 0.22 respectively). The data are presented as normalized area values relative to healthy control, p<0.001 or p<0.05 respectively) (Fig 6A–6D). These data suggest that anti-proNGF mAb reduces extravasation by either route of administration. Ameliorating vascular permeability also prevents extravasation of serum α_2_M and other inflammatory factors present in the serum, which would prevent further cytotoxic damage.

## Discussion

The treatment of retinal diseases poses a challenge due to the unique anatomy of the eye and the presence of the blood-retinal-barrier. Systemic, topical and intravitreal administration of drugs are used for different eye pathologies. Each approach has benefits and drawbacks, often very specific to the drug being tested. In addition to challenges associated with drug delivery, there is a scarcity of validated therapeutic targets and of therapeutic agents for treatment of neurodegenerative retinopathies.

Our study validates proNGF as a target, and the use of anti-proNGF mAbs delivered via systemic route or locally as a subconjunctival depot for therapy of DR, in addition to the lesser desirable intravitreal injection. The approach may be applied to other retinal neurodegenerative diseases such as glaucoma, where the proNGF/p75^NTR^ axis is implicated in disease etiology.

We evaluated the pharmacokinetics of anti-proNGF mAb in retinal tissues by three different routes of administration. Subconjunctival delivery of anti-proNGF antibody affording long–lasting therapeutic benefits represents a less invasive approach to modify disease through a promising novel target. We previously showed that an anti-proNGF mAb delivered IVT was therapeutic in DR as well [15]. IVT administration is an efficient method to assure delivery of a precise amount of drug to the vitreous and exposing the retina to the drug. This route requires relatively low doses and has limited systemic exposure. Hence, any toxicity is largely restricted to ocular tissues. However, IVT injections are relatively invasive, and repeated treatment can lead to serious side effects [24].

Specifically regarding Diabetes Retinopathy, current therapies include monthly IVT injections of anti-VEGF mAbs or traps, and anti-inflammatories [12]. Although efficient, these routes are invasive, costly and often carry serious complications. For example, an anti-VEGF mAb can cross the blood-retina barrier, but also inhibits serum VEGF, and prolonged anti-VEGF systemic treatment could induce adverse thromboembolic events [25]. Repeated IVT injections increase the risks of hemorrhage, endophthalmitis, retinal detachment and cataracts [26–28].

Additional targets are still sought for retinopathies [13], especially targets that are different from VEGF, or act upstream of VEGF [14]. Here, we provide an alternative target and route of administration. This information is relevant to our anti-proNGF mAb but also can be useful for improving delivery of other mAbs that are used for retinal pathologies.

Regarding systemic IV delivery, retinal and vitreous humor penetration by antibiotic and small lipophilic drugs are well studied [14, 29–31], but less is known about mAbs. The mAb must achieve therapeutic levels in the retina, overcoming the restrictive action of the blood-retina barrier, the dilutive effect of blood volume, and stability/clearance issues in circulation. Hence, higher doses are injected IV, leading to increased costs.

Generally IV administration of drugs is associated with systemic side effects (off target, as well as on-target when proNGF/p75^NTR^ are present in non-ocular tissues), and IV delivery can achieve low retinal concentrations due to systemic metabolism and the blood–eye barrier [32, 33]. However, in our study the administration of systemic anti-proNGF mAb in the diabetic mouse did not cause any detectable systemic toxicity, or effect on body weight yet it provided a long-lasting therapeutic effect in the retina.

Regarding SCJ, this route may afford retinal penetration through conjunctival and choroidal circulation [34]. Some studies have reported on antibody pharmacokinetics and eye tissue distribution after SCJ injections [35–39], suggesting that SCJ administration may achieve many drug delivery goals [36, 40]. Bevacizumab, a mAb against vascular endothelial growth factor (VEGF), can also penetrate tissues on the retina/choroid, iris/ciliary body, and vitreous via the sclera after SCJ injection [41, 42] [43]. We hypothesize that the anti-proNGF mAb may use the same pathway to reach the retina. The SCJ route limits distribution to the periocular region, requires less drug than IV–with lower costs–, and although systemic exposure is possible it is expected to happen at reduced or undetectable levels for our anti-proNGF mAb, especially when relatively low amounts are injected as a depot.

SCJ delivery is relatively less invasive than IVT and is considered a potential route for drug delivery to the posterior segment. Acute SCJ administration of anti-proNGF mAb achieved sufficient therapeutic concentrations in the retina to reduce edema, to lower production of neurotoxic cytokines TNFα and α_2_M, and maintained the neuronal retinal structure for prolonged periods. The benefit is comparable to IV or IVT injections of anti-proNGF.

In summary, a blocking anti-proNGF mAb given via the IVT, IV, or SCJ routes have similar kinetics and therapeutic efficacy in DR. It is remarkable that acute delivery of the mAb at 2.5 weeks after the onset of diabetes affords a long-term therapeutic effect for multiple pathological endpoints of DR, with efficacy lasting up to 10 weeks, long after clearance of the mAb. Most likely, inhibition of proNGF (preventing p75^NTR^ activation) resets the retinal environment to homeostasis. This notion would be consistent with reports that inhibition of the p75^NTR^ receptor results in similar long-lasting therapeutic effects in the STZ-mouse model of diabetic retinopathy [19] as well as other disease models [17–19].

Our work presents proNGF and its receptor p75^NTR^ as druggable targets that are disease-modifying. Given that proNGF expression and p75^NTR^ activation also play etiological roles in glaucoma, Retinitis Pigmentosa, and other chronic retinal diseases, it will be important to test the anti-proNGF mAb via the SCJ route in such disease models.

## Supporting information

S1 Fig Effect of systemic administration of anti-proNGF antibody on body weight. **(A)** Experimental paradigm and endpoints of mouse STZ-induced diabetic model. Mice were injected with STZ for five consecutive days starting on day 0. At 2.5 weeks, the mice were treated with vehicle (PBS) or anti-proNGF. Analyses were done starting from week 3 up to week 10. **(B)** Average body weights ± SEM relative to week 2 (one-way ANOVA, followed by Bonferroni post-hoc analysis, n = 4 mice per group). Mice body weights were measured weekly. Body weight progression was analyzed by normalizing the body weight of each group to their weights at week 2. This approach counteracts the variability of the weight loss that is induced by diabetes, prior to drug treatments.
(TIF)

S2 Fig Characterization of biotinylated anti-proNGF mAb. Characterization of anti-proNGF•biotin mAb (20 ng) under reducing or non-reducing conditions yield the expected 150 kDa, or the 57 kDa and 25 kDa bands in SDS-PAGE. The biotinylation procedure was performed using NHS-Biotin (Pierce).
(TIF)
