# Supplementary figures and images for: The route of administration influences the therapeutic index of an anti-proNGF neutralizing mAb for experimental treatment of Diabetic Retinopathy
Source: PLoS One. 2018 Jun 21;13(6):e0199079. doi: 10.1371/journal.pone.0199079 (PMC6013198; doi:10.1371/journal.pone.0199079)

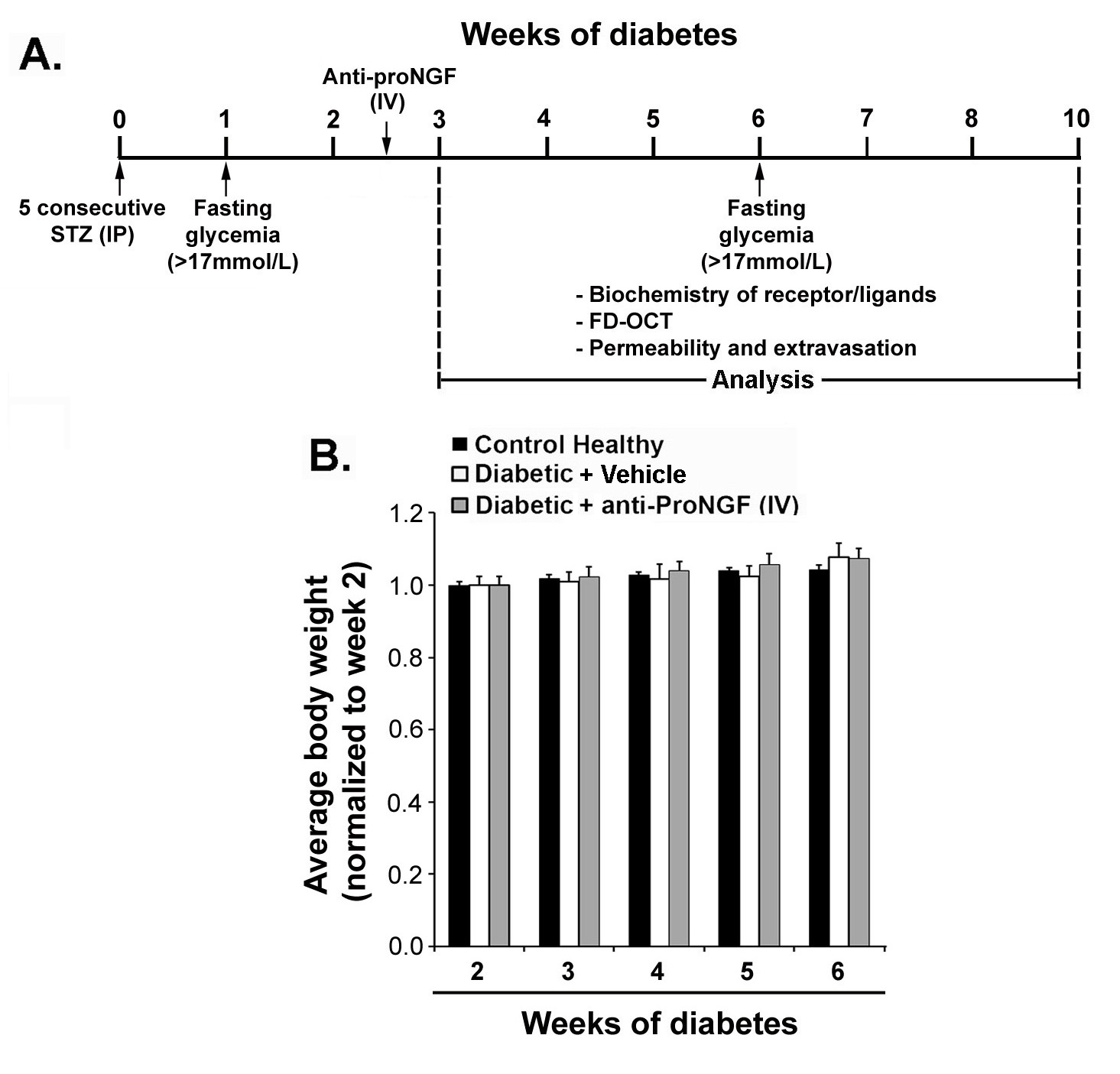

Supplement: S1 Fig — (A) Experimental paradigm and endpoints of mouse STZ-induced diabetic model. Mice were injected with STZ for five consecutive days starting on day 0. At 2.5 weeks, the mice were treated with vehicle (PBS) or anti-proNGF. Analyses were done starting from week 3 up to week 10. (B) Average body weights ± SEM relative to week 2 (one-way ANOVA, followed by Bonferroni post-hoc analysis, n = 4 mice per group). Mice body weights were measured weekly. Body weight progression was analyzed by normalizing the body weight of each group to their weights at week 2. This approach counteracts the variability of the weight loss that is induced by diabetes, prior to drug treatments. (TIF) [file pone.0199079.s001.tif]

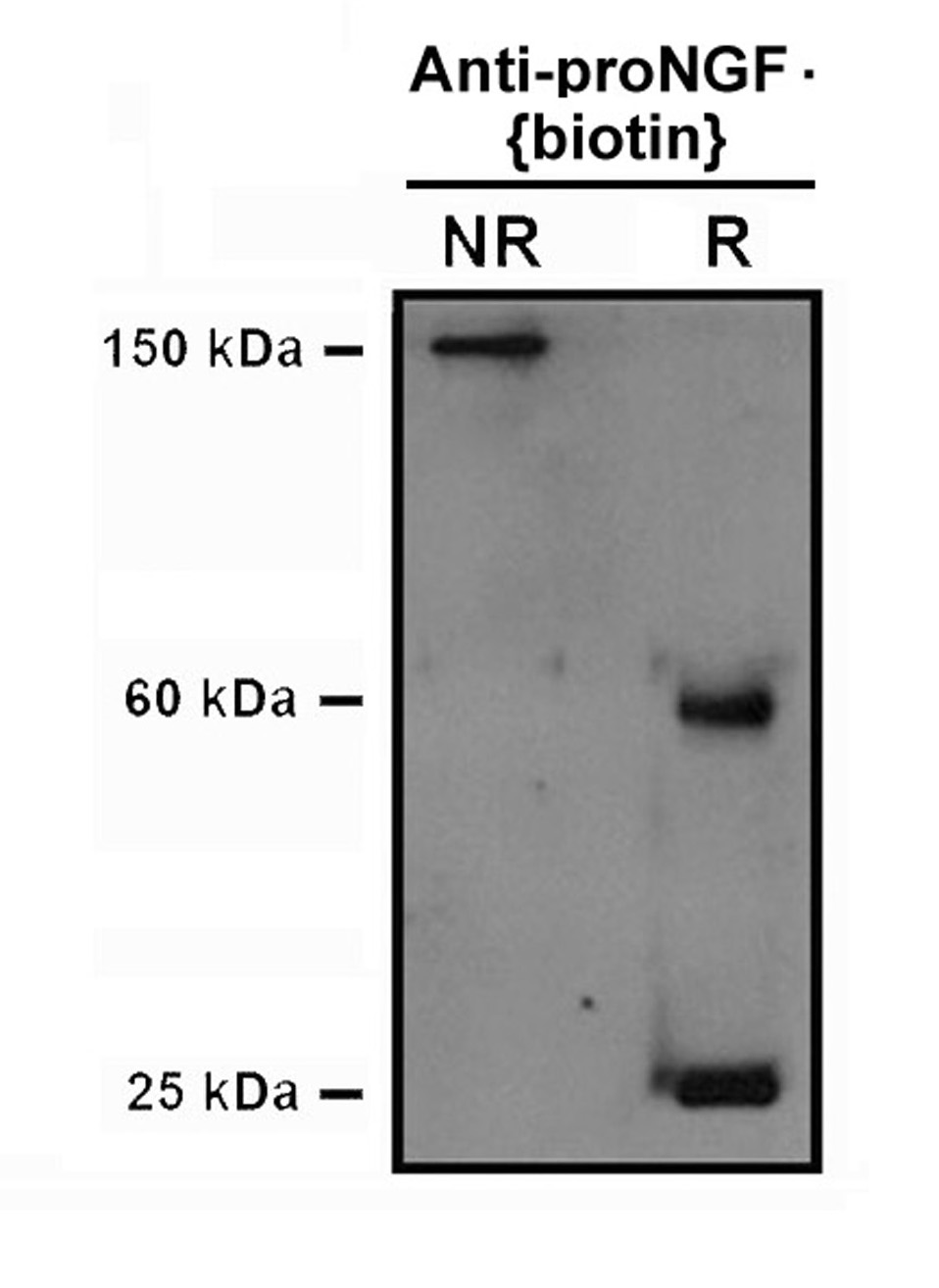

Supplement: S2 Fig — Characterization of anti-proNGF•biotin mAb (20 ng) under reducing or non-reducing conditions yield the expected 150 kDa, or the 57 kDa and 25 kDa bands in SDS-PAGE. The biotinylation procedure was performed using NHS-Biotin (Pierce). (TIF) [file pone.0199079.s002.tif]
